# Relationship between Σ3 Boundaries, Dislocation Slip, and Plasticity in Pure Nickel

**DOI:** 10.3390/ma16072853

**Published:** 2023-04-03

**Authors:** Yao Lin, Luyi Han, Guangchun Wang

**Affiliations:** Key Laboratory for Liquid-Solid Structural Evolution and Processing of Materials, Ministry of Education, Shandong University, Jinan 250061, China

**Keywords:** crystal plastic finite element simulation, dislocation slip, Σ3 boundary, plastic deformation, quasi in situ tensile test

## Abstract

This study investigated the relationship between the Σ3 boundaries, dislocation slip, and plasticity in pure nickel wires after grain boundary (GB) modification. Both quasi in situ tensile tests and simulations were employed. During plastic deformation, twins surrounded by Σ3 boundaries may exhibit a good deformation coordination. With an increase in strain, the slip systems corresponding to the maximum Schmid factor and the actual activated slip systems remain unchanged. Even sub-grains can maintain the dominant slip system of their origin matrix grains. Slip systems with slip planes (111) and (1−1−1) are the most active. Moreover, random boundaries have strong hindering effects on dislocations, and the nearby stress accumulates continuously with an increase in strain. In contrast, Σ3 boundaries demonstrate weak blocking effects and can release the nearby stress due to their unique interfacial structures, which is favorable for improving plasticity. They are more penetrable for dislocations or may react with the piled dislocations. In addition, some Σ3 boundaries can improve their geometrical compatibility factor with an increase in the strain, which enhances the deformation coordination of the grains. The research results provide a better understanding of the plasticizing mechanism for face-centered cubic (fcc) materials after grain boundary modification.

## 1. Introduction

Grain boundaries (GBs) are important microscopic features of metallic materials. The GB type and distribution significantly affect the physical and chemical properties of materials. Therefore, the GB-related properties of materials can be improved by adjusting the GB structure and characteristics. Since the concept of “GB engineering” was proposed by the Japanese scientist Tadao Watanabe [1] in 1984, GB modification has been extensively studied in the laboratory. The modification processes and mechanisms of different materials are different. For face-centered cubic (fcc) metals and alloys, such as nickel and copper, GB modification is primarily based on annealing twins and is achieved by increasing the content of twins. The misorientation relationship between the majority of annealing twins and matrix grains is typically <111>/60°. Such a relationship can be determined as Σ3 according to the coincidence site lattice (CSL) model [2,3]. Most annealing twins in fcc materials have Σ3 boundaries.

Σ3 boundaries occupy the majority of low-ΣCSL boundaries (also called special boundaries) and significantly affect the material properties. Previous studies have shown that the energy and mobility of special boundaries, particularly Σ3 boundaries, are low. This phenomenon has a significant inhibitory effect on the GB corrosion, segregation, and fracture. In contrast, random boundaries with higher GB energies and mobilities are more likely to generate microcracks and accelerate the crack propagation [4]. Some researchers believe that the specific topological structure and crystallographic characteristics of annealing twin boundaries can increase the potential of slip band initiation [5]. The plasticity can also be improved because special boundaries can result in a more uniform strain distribution during plastic deformation. Studies on fcc metals indicate that cross-slip can reduce the tip stress and activate intergranular plasticity [6]. On this basis, researchers have improved the plasticity of metals by adjusting the proportion and distribution of special boundaries and random boundaries, and the failure behavior resulting from poor plasticity during the application process can be reduced. In the studies employing pure nickel [7,8,9,10] and 316 stainless steel [11], the material elongation was observed to increase with a fraction increase in special boundaries in specific cases. Thus, improving material plasticity via GB modification is highly feasible.

At present, extensively detailed data on the plasticizing parameters are available for GB modification. Many studies have characterized the effects of GB modification and investigated its deep plasticizing mechanisms from various aspects. Common microscopic indices for characterizing plasticity include the Schmid and geometrical compatibility factors, which represent the degree of difficulty of the intragranular slip and slip-twinning transmission through GBs, respectively. Explorations about plasticizing mechanisms primarily focus on the overall influence of various microscopic structures on properties such as the recrystallization texture, dislocation density, and Taylor factor [11,12,13,14,15] or analyzing the effect of the length and quantity ratio of all the special boundaries [16,17,18,19,20]. However, the microscopic essence of plastic deformation is the dislocation slip and twinning. The interaction between the GBs and dislocations in this complex process must be clarified and is worthy of further discussion.

The plastic deformation of polycrystals includes intracrystalline and intergranular deformations. The fcc metal has 12 groups of slip systems, and each slip plane has three slip directions. Therefore, it has a good plastic deformation basis. However, plastic deformation cannot proceed indefinitely because the dislocation slip is hindered by the GBs or other phases, resulting in stress concentration and, ultimately, material failure [6,21]. The interaction mechanisms of dislocations with twin boundaries or random boundaries are very complex and have been explored by nanoscale experiments and molecular dynamics simulations. The hindrance effect of GBs is significant, and different types of GBs have different hindrance effects on the dislocations [16,22]. Dislocations might dissociate and slide at the Σ3 boundaries, depending on the specific conditions, such as the energy barrier of the materials [23,24]. It was found that the flow stress of the Σ3 boundary would influence the dislocation slip transmission [25]. A screw dislocation, of which the Burgers vector was parallel to the Σ3 boundary, was more necessary to be transmitted across the boundary; Then, the Σ3 boundary might generate a new screw dislocation, of which the Burgers vector was perpendicular to the Σ3 boundary [26]. In regard to the random boundaries, the dislocation densities changed significantly during plastic deformation, as the dislocations were emitted from and absorbed at the random boundaries [27]. Most of this research has focused on specific dislocations with deformation twins or nanotwins [28,29]. However, the twins are basically annealing twins in fcc metals after GB modification. Moreover, plastic deformation involves numerous dislocations, even within one grain. It is necessary to pay attention to the behavior of the interactions between the dislocations and grain boundaries in a more macroscopic view. Therefore, studying the relationship between various types of GBs with dislocations, crystal orientation, and the plastic deformation mechanisms of fcc metals on the grain-to-grain level is of considerable significance for optimizing their practical applications.

In this study, a pure nickel wire undergoing GB modification was selected for the quasi in situ tensile test. The crystal orientation, slip system activation, and relationship between the dislocation and GB were investigated comprehensively. Crystal plastic finite element (CPFE) simulations were used to explore the relationship between the Σ3 boundary, dislocation slip, and material plasticity in pure nickel and explain the plasticization mechanism via GB modification.

## 2. Materials and Methods

### 2.1. Quasi In Situ Tensile Test

**Figure 1:**
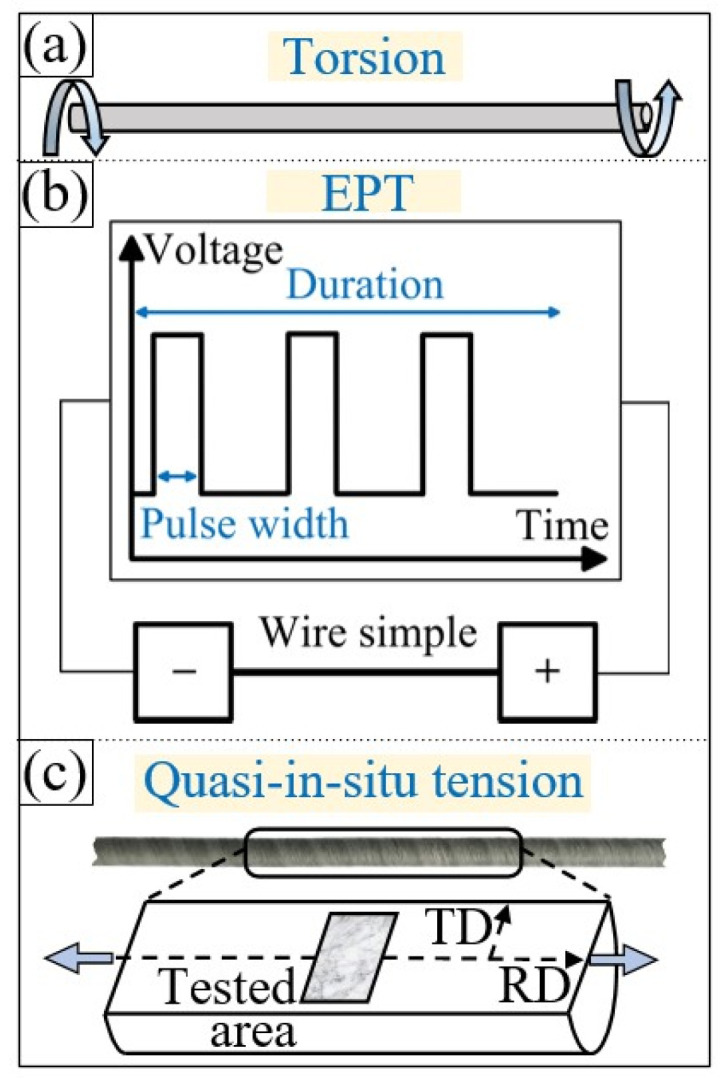
Schematic diagram of the (**a**) torsion, (**b**) electrical pulse treatment, and (**c**) selected position for micro observation in quasi in situ tensile test.

To study the plastic deformation mechanism of materials with a high proportion of special boundaries, a pure nickel (Ni200) wire with a diameter of 1 mm was modified via torsion and electric pulse treatment to induce numerous special boundaries. Figure 1 shows a schematic diagram of the torsion, electrical pulse treatment, and quasi in situ tensile test of the nickel wire sample. According to a previous study [30], the proportion of special boundaries can reach 55.1% after the nickel wire is subjected to a unidirectional torsion of 90 r and electrical pulse treatment with a current density of 1900 A/mm^2^, frequency of 200 Hz, and duration of 120 s. The fracture elongation obtained after the tensile test (2 mm/min) at 25 °C is up to 44.7%, which is 17.5% higher than that of an untreated sample.

The middle section (length: 13 mm) of the modified sample was cut off, and the longitudinal section was subjected to mechanical polishing and electropolishing. The in situ tensile test module (MZ0-1) was employed for the quasi in situ tensile testing (10 μm/s). Scanning electron microscopy (SEM; Tescan Lyra3) was used to observe the microstructural evolution of the selected region in the longitudinal section during the tensile process, as shown in Figure 1c. The Channel 5 (5.0.9.0) software and the MATLAB MTEX crystal analysis toolbox were used to analyze the crystal orientation information obtained via electron backscatter diffraction (EBSD).

### 2.2. CPFE Simulation Based on the Phenomenological Model

To investigate the quasi in situ tensile process of the pure nickel wire further, a continuity analysis of its plastic deformation behavior was conducted using the open-source software DAMASK [31,32]. According to the phenomenological constitutive law of crystal plasticity [33,34], the shear rate ${\dot{\gamma }}^{\alpha }$ on the slip system *α* is a function of the critical resolved shear stress $\tau_{c}^{\alpha }$ and resolved shear stress *τ^α^* for the slip system *α*:

(1) $${\dot{\gamma }}^{\alpha }={\dot{\gamma }}_{0}{\left|\frac{\tau^{\alpha }}{\tau_{0}^{\alpha }}\right|}^{n}\mathrm{sign}(\tau^{\alpha })$$

where ${\dot{\gamma }}_{0}$ is the reference shear-strain rate, and *n* is the stress exponent. The influence of any slip system *β* on the hardening behavior of the slip system *α* is as follows:

(2) $${\dot{\tau }}_{0}^{\alpha }\;=\sum_{\beta =1}^{N}h_{\alpha \beta }\left|{\dot{\gamma }}^{\beta }\right|$$

where *h_αβ_* is the hardening matrix that empirically illustrates the micromechanical interactions among various slip systems.

(3) $$h_{\alpha \beta }=q_{\alpha \beta }[h_{0}{\left(1-\frac{\tau_{0}^{\alpha }}{\tau_{\infty }}\right)}^{a}]$$

where *h*_0_, *a*, and $\tau_{\infty }$ represent the self-hardening coefficient, hardening exponent, and saturation value of the slip resistance, respectively. Moreover, *q_αβ_* is the latent-hardening coefficient.

The material parameters employed in the crystal plastic model were set according to the relevant literature [31,32,35,36]. The anisotropic elastic properties for nickel were set as *C*_11_ = 251.0 MPa, *C*_12_ = 150.0 MPa, and *C*_44_ = 123.7 MPa. The constants in the hardening law were selected to characterize the plastic behavior of the crystals. The initial slip resistance *τ*_0_ was 26.1 MPa, the saturation slipping resistance *τ*_∞_ was 240 MPa, the stress exponent *n* was 83.3, the reference strain rate ${\dot{\gamma }}_{0}$ was 1 × 10^−3^ s^−1^, the initial hardening modulus *h*_0_ was 365 MPa, and the hardening ratio *a* was 1.

## 3. Results and Discussion of Quasi In Situ Tensile Test

### 3.1. Variations of GB Characteristics

**Figure 2:**
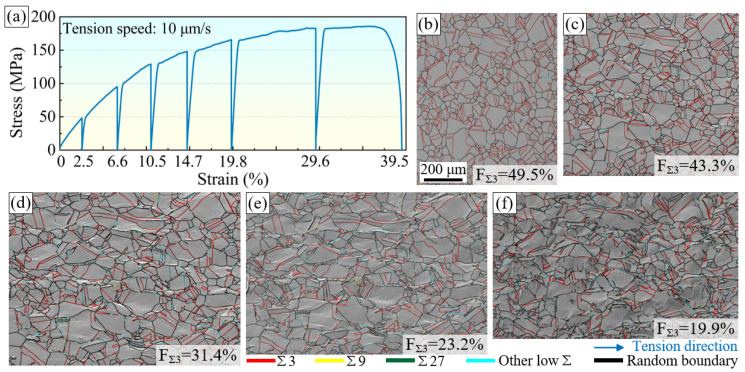
(**a**) Stress–strain curve of the nickel wire during quasi in situ tensile test, (**b**–**f**) SEM and GBCD diagrams at strains of 0, 10.5%, 19.8%, 29.6%, and 39.5%, respectively.

The microstructural evolution during plastic deformation was studied via quasi in situ tensile testing of the nickel wire, which contained a sufficient number of special boundaries after GB modification. Figure 2a shows the stress–strain curve of the nickel wire during the quasi in situ tensile test, and Figure 2b–f show the SEM and GB characteristic distribution (GBCD) diagrams at strains of 0, 10.5%, 19.8%, 29.6%, and 39.5%, respectively. The SEM diagrams show that the originally flat surface of the sample becomes uneven during the tensile process and that an orange-peel effect occurs. With an increase in the tensile strain, the observed region is elongated along the tensile direction. Clear and orderly slip traces can be observed in some grains. During this process, the grains also elongate gradually. However, most of them retain their original morphology. At high strains, a few grains are destroyed, owing to the incoordination of the deformation. The phenomenon of the formation of sub-GBs within large grains and fragmentation to form sub-grains is then observed.

The GBCD also varied during the plastic deformation. Figure 2b shows that the initial sample contains many uniformly distributed Σ3 boundaries. They are annealing twin boundaries generated during GB modification. Σ3 boundaries account for 49.5% of the high-angle boundaries, effectively breaking the connectivity of the random boundary networks. This is generally considered favorable for material plasticity [4,37]. However, the proportion of Σ3 boundaries among the high-angle boundaries decreased significantly during the tensile process. It decreased to 19.9% when the sample broke. Moreover, it can also be seen from Figure 2f that many preserved Σ3 boundaries are fragmentarily distributed in the random boundary network. The random boundaries increase significantly during the tension process and show a dense state of aggregated distribution in some regions. This may be one of the reasons for the decrease in the fraction of Σ3 boundaries.

### 3.2. Evolution of Crystal Orientation

**Figure 3:**
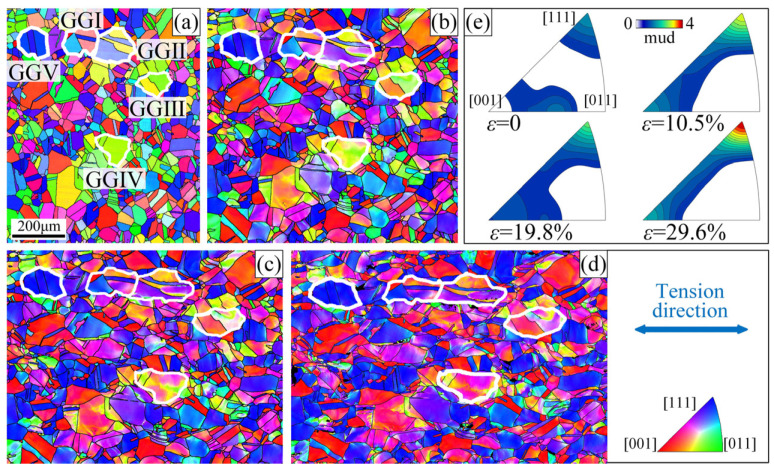
(**a**–**d**) OIM at strain values of 0, 10.5%, 19.8%, and 29.6%, respectively. (**e**) Inverse pole figures along RD direction at strain values of 0, 10.5%, 19.8%, and 29.6%, respectively.

Figure 3a–d show the orientation imaging micrograph (OIM) at strain values of 0, 10.5%, 19.8%, and 29.6%, respectively. Figure 3e shows the corresponding inverse pole figures along the RD direction. When the tensile strain was 39.5%, the severe deformation resulted in a low indexing rate of EBSD and serious errors in the orientation identification. Therefore, this state was not analyzed in detail. As shown in Figure 3, the orientation distribution is relatively uniform in the initial strain-free condition, with slightly preferred orientations [111] and [012], which can be considered to be the recrystallization texture formed during GB modification. However, with an increase in strain, the preferred orientation of [012] rapidly disappears, the deformation texture of [111] continuously strengthens, and a weak deformation texture of [001] appears. This indicates that the grains have apparent [111] + [001] preferred orientations during the stretching process, which are the common fiber textures in fcc metals.

**Figure 4:**
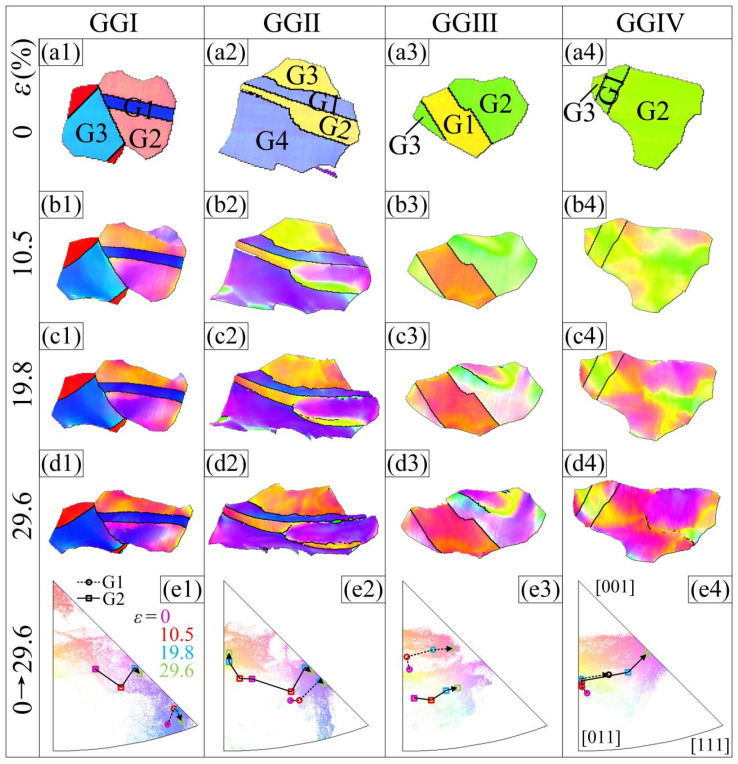
(**a1**–**a4**) OIM of grain groups GGⅠ~GGⅣ at strain values of 0. (**b1**–**b4**) OIM of grain groups GGⅠ~GGⅣ at strain values of 10.5%. (**c1**–**c4**) OIM of grain groups GGⅠ~GGⅣ at strain values of 19.8%. (**d1**–**d4**) OIM of grain groups GGⅠ~GGⅣ at strain values of and 29.6%. (**e1**–**e4**) Inverse pole figures along RD direction and the traces of pole point positions of grains G1 and G2 when the strain increased from 0 to 29.6%.

To further study the variation in the crystal orientation during tensile deformation and its relationship with the Σ3 boundaries, four grain groups, GGⅠ–GGⅣ, were selected in the observation area, as shown by the white lines in Figure 3a–d. In the OIMs, different colors represent different orientations. With an increase in strain, the coordinated rotation of the grains results in a variation in the orientation. Almost all of the grains rotate toward the [001]–[111] line link and continue to rotate in the [111] direction. However, the rotation amplitudes of the grains with different initial orientations are not the same. In Figure 4, the grains in the grain group are numbered, and the inverse pole figures show the traces of the pole point positions of grains G1 (circle + dashed line) and G2 (box + solid line) when the strain increases from 0 to 29.6%. All of the grains numbered G1 in the figures are annealing twins in the strain-free state with a boundary of Σ3. The initial orientation of G1 in GGⅠ is near the [111] direction, which only slightly change during the deformation process. The change in the G1 orientation in GGⅡ and GGⅣ is also small. However, the orientation of G1 in GGⅢ varies significantly from [012] to approximately [−239]. The pole point trace of the twin G1 can also prove this. In addition, for the G1 twins, the total trace of the pole points is shorter than that of the adjacent general G2 grains, indicating that the rotation amplitude is smaller. Simultaneously, it also shows that even inside the twins, the orientation changes during plastic deformation; that is, the twins can also rotate. However, the rotation amplitude is smaller than that of the non-twin grains.

TThe macroscopic plastic deformation of polycrystals is achieved via continuous deformation and coordinated rotation of grains. When a grain is severely constrained by the surrounding grains or is in a hard orientation, it is difficult to rotate, which can easily stimulate microcracks and result in material failure. At this time, if the grain is supposed to continue plastic deformation, it might break into sub-grains to increase the freedom of deformation. Grain G2 in grain group GGⅡ (Figure 4(b2)) is yellow on the left side and purple on the right side at a strain of 10.5%, indicating an apparent orientation gradient and a tendency to form sub-GBs. At this time, in the inverse pole figure in Figure 4(e2), the pole point with the highest intensity of this grain is classified into two (as shown by red squares), one toward the [001]–[011] line link and the other toward the [001]–[111] line link. This orientation gradient gradually increases with the increasing strain. In the orientation imaging micrograph (OIM), the colors of the crystals represent their orientation. At a strain of 29.6%, the left side of the grain is primarily orange (near the [013] direction) and the right side is primarily purple (near the [112] direction). The sub−GB between these two parts evolves into a high-angle random boundary. In the GBCD diagram of the sample after fracture (Figure 2f), these two sub-grains are completely separated into two grains. Similar phenomena can be observed in other non-twin grains. However, they are rare in twins surrounded by Σ3 boundaries, either because twins are typically smaller than ordinary grains or because such grains within the Σ3 boundaries have better deformation coordination with the adjacent grains.

### 3.3. Analysis of the Activated Slip System

**Figure 5:**
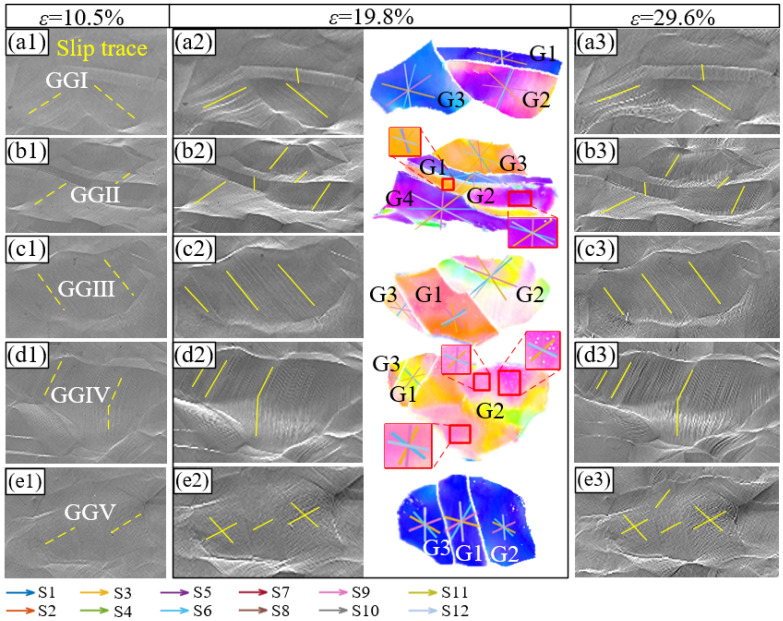
SEM and OIM of grain groups (**a1**–**a3**) GGⅠ, (**b1**–**b3**) GGⅡ, (**c1**–**c3**) GGⅢ, (**d1**–**d3**) GGⅣ, and (**e1**–**e3**) GGⅤ at strain values of 10.5%, 19.8%, and 29.6%, respectively. The yellow lines in SEM diagrams are slip traces on the surface of the sample. The colored lines in OIM represent the different slip systems in grains. The G1, G2, G3, G4 are the numbers of corresponding grains.

When the crystal is under stress, the spatial orientations with external force are different for different slip systems, and the shear stress components are also different. When the shear stress reaches the critical value of a slip system, slipping begins to occur. For a single grain, the larger the Schmid factor of a slip system, the larger the shear stress, and the earlier the slip occurs. Several grain groups in the observation region were selected for further analysis. As shown in Figure 2b, the initial surface of the sample is flat. At a tensile strain of 10.5% (Figure 5(a1–e1)), slip traces begin to appear on the surface of the sample. However, the slip traces are fuzzy because of the small deformation, and the dotted lines in the figure indicate their directions. When the strain increases to 19.8% (Figure 5(a2–e2)), neatly arranged slip traces are observed in most of the grains, and their directions are marked with solid lines in the figure. At the strain of 29.6%, the degree of surface concavity is intensified. However, the distribution and direction of the slip traces are consistent with those of the strain at 19.8%, indicating that the type of slip system activated in the grain is unchanged during this process. In addition, the slip bands in Figure 2 and Figure 5 indicate that, regardless of the strain, crystals are mostly dominated by the single slip.

Using the MTEX toolbox, we calculated the corresponding Schmid factors of 12 slip systems under different strain conditions in the observed area. With an increase in strain, the corresponding slip system of the maximum Schmid factor of each grain remained unchanged. This again shows that the active slip system in the grains was stable during plastic deformation. Table 1 shows the absolute value of the Schmid factors at a strain of 19.8%, and the maximum Schmid factor corresponding to each grain is shown in bold. In the OIMs of a single grain, the slip plane of each slip system is marked with lines of a different color (Figure 5). Notably, because the slip planes of slip systems S1–S3 are identical, their corresponding lines in the figure coincide, as do the other slip systems. The slip traces in the SEM images were compared with the calculated slip planes in the OIM images to identify the slip plane type. Subsequently, combined with the Schmid factors corresponding to different slip directions with the same slip plane, the actual activated slip systems were determined, and the corresponding Schmid factors were marked in blue. Among the 13 grains listed in Table 1, the actual activated slip systems of ten grains are consistent with those corresponding to their maximum Schmid factors, respectively. Moreover, 60% of all the activated slip systems are S1–S3, whereas 33% are S7–S9. In addition to the grains listed in Table 1, 15 grains in the observation region were randomly selected to analyze the slip system types. The results showed that S1–S3 and S7–S9 occupied the majority. In other words, S1–S3 with slip planes (111) and S7–S9 with slip planes (1−1−1) were the most active slip systems. Notably, two slip systems are activated simultaneously in some grains, such as GGⅡ−G2 and GGⅣ−G2. Both Schmid factors of the two activated slip systems are large. In fcc nickel with good plasticity, although the actual activated slip system does not necessarily have the largest Schmid factor, a high Schmid factor is conducive to the initiation of the slip system. This is because other factors, such as the GBs, can also affect the transformation of the dislocations within and between grains, which influences the function of the Schmid factor to some extent.

To characterize the interaction between the GBs and dislocations, a geometrical compatibility factor (*m*′) was introduced. The compatibility factor is determined by the slip plane and the direction of the adjacent grains, and its value ranges between zero and one. When the value is zero, the slip transmission is completely hindered by the GB, and when the value is one, the slip can completely cross the GB. In other words, the larger the value, the more favorable the plastic deformation. Studies have shown that *m*′ can affect the activation of the slip systems. Therefore, we selected the grains whose actual activated slip system was inconsistent with the maximum Schmid factor in Table 1 and calculated the corresponding *m*′ values, which are shown in Table 2. According to Figure 5(b2) and Table 1, the actual activated slip system in grains GGⅡ–G3 belongs to S1–S3, and the maximum Schmid factor corresponds to slip system S8. Compared with Table 2, the *m*’ values between slip systems S1–S3 and S8 of grain G3 with the neighboring grain G1 are 0.003, 0.475, 0.478, and 0.400, respectively. The slip system S2, with both a large Schmid factor and *m*′, is determined to be the actual active slip system. However, notably, the maximum *m*′ between grains G1 and G3 corresponds to slip system S9. This indicates that *m*′ also affects the initiation of slip systems. However, its effect is smaller than that of the Schmid factor. Similarly, the highest and second-highest Schmid factors of grain GGⅣ−G2 correspond to slip systems S2 and S8, respectively, and the maximum *m*′ values correspond to slip systems S2 and S3, respectively. However, the actual slip traces indicate that double slip is activated in this grain, namely S2 and S8. This analysis shows that the Schmid factor and *m*′ jointly determine the activated slip system and that the Schmid factor plays the predominant role.

**Figure 6:**
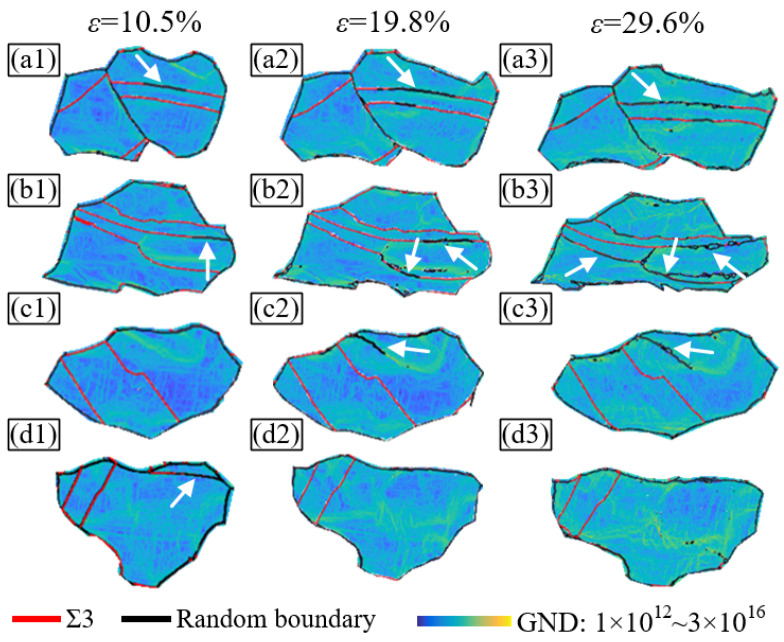
The GND distribution of grain groups (**a1**–**a3**) GGI, (**b1**–**b3**) GGII, (**c1**–**c3**) GGIII, and (**d1**–**d3**) GGIV at strain values of 10.5%, 19.8%, and 29.6%, respectively.

Although *m*′ has little effect on the activation of the slip system, it can still quantitatively characterize the hindrance effect of various GBs on the dislocation slip. Combined with the GBCD diagrams in Figure 2, the variation in the *m*′ values of the Σ3 boundaries in the plastic deformation process was further explored. Some *m*′ values of the Σ3 boundaries remained unchanged and some increased or decreased. Several grains were selected and the variation of their *m*′ values during the tensile test was calculated, as listed in Table 3. In grain group GGⅡ, the activated slip systems of grain G2 are S2 and S9, and their corresponding *m*′ values in the initial state are 0.464 and 0.638, respectively. When the strain increases to 10.5%, the right side of a Σ3 boundary no longer satisfies the Brandon criterion and is recognized as a random boundary segment (Figure 6), and the *m*′ values corresponding to S2 and S9 decrease to 0.461 and 0.592, respectively. With the increasing strain, the corresponding *m*′ continues to decrease. However, it should be noted that, in this case, it is not necessarily the type of transition of the grain boundary. Identification errors due to dislocation pile-ups near the Σ3 boundary should also be considered. The SEM image shows that grain G1 is crushed against G2 and gradually upwarps at a specific angle, indicating that the deformation here is uncoordinated. This proves that the Σ3 boundary has a higher *m*′ and shows a slight obstacle effect compared with the random boundaries. More Σ3 and random boundaries in the observation area were randomly selected to calculate *m*′, and the results remained the same. In addition, some Σ3 boundaries were stable throughout the whole deformation process. For example, the GBs between G1 and G2 in grain group GGⅢ, whose *m*′ remained above 0.960 and were stable throughout the deformation process. No upwarp was observed between these two grains, indicating good dislocation transitivity. In addition, some Σ3 boundaries not only maintained a good mirror symmetry relationship, but also resulted in an increase in the corresponding *m*′ values with the increasing strain. For example, the *m*′ between grains G1 and G2 in grain group GGⅤ increased from 0.477 to 0.552 with an increase in the strain from 0 to 29.6%, and the blocking effect of the dislocations gradually decreased. Similarly, no warping occurred between the two grains. Thus, Σ3 boundaries are more conducive to dislocation slip transformation and deformation coordination than random boundaries, and a high proportion of Σ3 boundaries positively affects plastic deformation.

### 3.4. Influence of GBs on Dislocation Density

To investigate the relationship between the GB and dislocations, the distribution and density of the geometrically necessary dislocations (GNDs) in the observation area were calculated using MTEX and self-written programming at strains of 10.5%, 19.8%, and 29.6%, respectively. The GND distribution of grain groups GGⅠ–GGⅣ is shown in Figure 6. As seen in Figure 6, the GND density increases significantly with the accumulation of strain. However, its distribution within and among the grains is nonuniform. This is because GNDs accumulate near the GBs and within some grains. In addition, the density of the GNDs near the random boundaries is higher, whereas it is lower near the Σ3 boundaries. This indicates that dislocations are more likely to pile-up at random boundaries than at Σ3 boundaries. Alternatively, Σ3 boundaries are significantly less likely to impede the dislocation slip than random boundaries. The slip traces in the SEM diagrams (Figure 5) show that the actual activated slip systems of the two grains separated by twins are still the same, such as G2 and G3 in GGⅡ and G2 and G3 in GGⅢ. The twins between these separated grains also show slip traces in the same direction, even though the twins have completely different crystal orientations from these separated grains. This demonstrates that twins or Σ3 twin boundaries can transfer dislocations effectively. The underlying mechanisms are discussed in detail in Section 4.2. Combined with the OIMs in Figure 4, many band-like areas exhibit high GND densities, of which the orientations on both sides have noticeable differences (shown by the white arrow). However, the direction of their slip traces is the same. This indicates that in the plastic deformation process of pure nickel, the initial slip system in a grain will always dominant, and it is difficult to trigger multiple slips or cross-slips. Even if large grains split into sub-grains with completely different orientations, the sub-grains can still maintain the dominant slip system of the matrix grains during subsequent deformation.

## 4. Results and Discussion of CPFE Simulation

**Figure 7:**
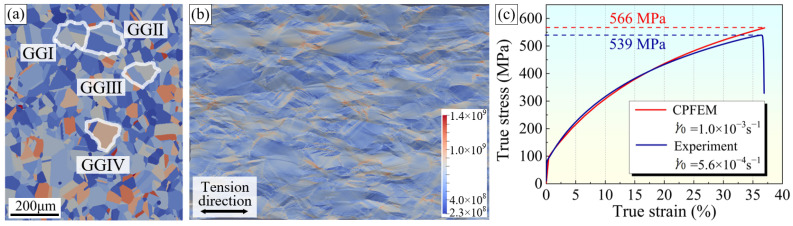
(**a**) The initial state of CPFE simulation and positions of grain groups. (**b**) The grain morphology and stress distribution in the simulated final state. (**c**) The true stress–strain curves acquired form the CPFE simulation and standard tensile test.

The variations in stress and strain near random and Σ3 boundaries were thoroughly investigated via CPFE simulation to verify the role of various types of GBs in material plasticity. The crystal orientation information at a strain value of 0 in the quasi in situ EBSD test was imported into the simulation software as the initial state of the CPFE simulation, as shown in Figure 7a. Figure 7b shows the grain morphology and stress distribution in the simulated final state (engineering strain of 40%). The grain morphology variations correspond well with the results of the quasi in situ tensile test. In Figure 7c, the red line shows the simulated true stress–strain curve. The blue curve is acquired from the uniaxial tensile test at room temperature of the sample subjected to the same GB modification. These two curves are consistent, indicating that the simulation model and parameters are accurate and that the simulation results are reliable. Notably, although the strain rates adopted by the two curves are slightly different, the plastic deformation behavior of pure nickel is not sensitive to the strain rate in the range used. Therefore, the effect of the strain rate is negligible.

### 4.1. Stress Concentration near Different Types of GBs

**Figure 8:**
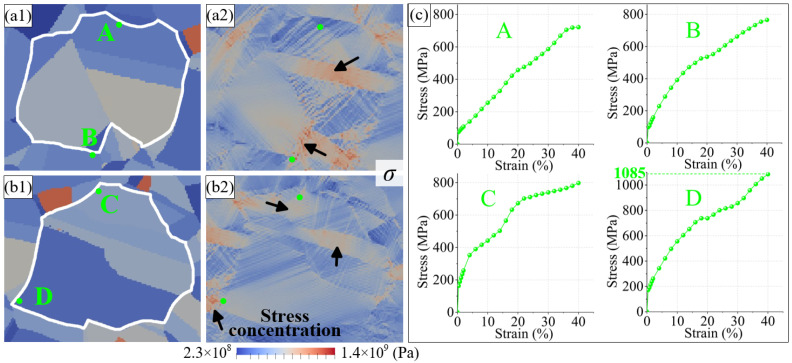
The (**a1**) position of points near random boundaries at initial simulation state and (**a2**) stress distribution at final simulation state in grain group GGⅠ. The (**b1**) position of points near random boundaries at initial simulation state and (**b2**) stress distribution at final simulation state in grain group GGⅡ. (**c**) The stress variation of points A–D.

The stress variation near the random boundaries during plastic deformation was first analyzed. Figure 8 shows the stress distribution in grain groups GGⅠ and GGⅡ after 40% stretching, respectively. Severe stress concentrations develop within or near the GBs of some grains, as indicated by the black arrows in the figures. The stress distribution is bounded by the GB, which shows the inhomogeneity of the plastic deformation attributed to the GB. In Figure 8a,b, A–D are four points near random boundaries, and their corresponding stress–strain curves are shown in Figure 8c. As shown in the figure, the stress at points A–D increases gradually during the entire plastic deformation process, and the stress at point D increases to 1085 MPa, indicating that the deformation coordination at this point is extremely poor. If the applied stress continues, the crystal continuity is difficult to maintain, and the initiation, as well as rapid expansion, of intergranular microcracks will be easily induced. Therefore, the presence of random boundaries increases the possibility of fracture failure.

**Figure 9:**
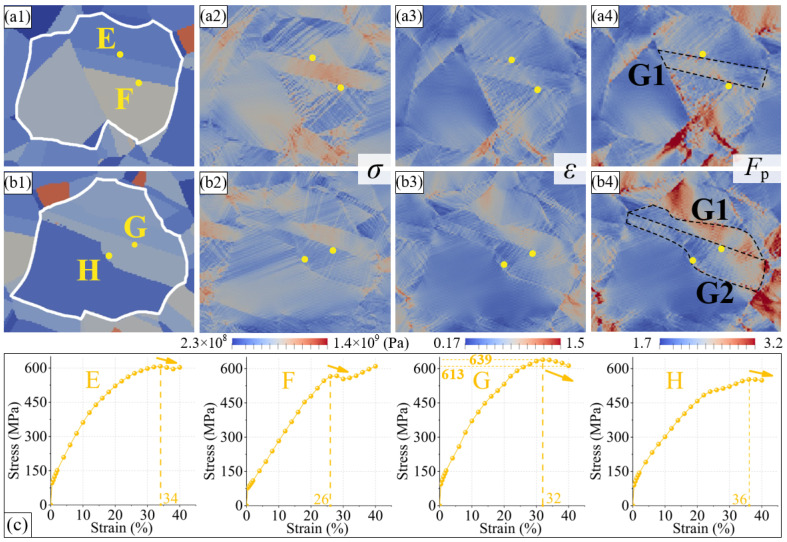
(**a1**) The position of points near Σ3 boundaries at initial simulation state in grain group GGⅠ. (**a2**–**a4**) The distribution of stress, strain, and plastic deformation gradient at final simulation state in grain group GGⅠ. (**b1**) The position of points near Σ3 boundaries at initial simulation state in grain group GGⅡ. (**b2**–**b4**) The distribution of stress, strain, and plastic deformation gradient at final simulation state in grain group GGⅡ. (**c**) The stress variation of points E–H.

The stress variations near the Σ3 boundaries were different. As shown in Figure 6, some Σ3 boundaries exceed the Brandon criterion during the plastic deformation process, and the dislocation density around them decreases significantly, suggesting an alleviation of the stress concentration. In the CPFE simulation, the stress data of the points near the Σ3 boundaries were extracted directly for quantitative analysis. Figure 9(a1–a4) shows the stress and strain distributions of grain group GGⅠ at the final state (tensile strain of 40%). Points E and F are close to the Σ3 boundary of twin G1 and are located outside the twin. The variation in the stress at these two points, with the tensile strain, is shown in Figure 9c. The stress values at points E and F show a downward trend at a 34% and 26% strain, respectively, and then gradually increase, finally reaching approximately 610 MPa. At the end of the stretching process, both the strain and plastic deformation gradients at these two points are relatively low. In grain group GGⅡ (Figure 9(b1–b4)), G and H are two points close to the Σ3 boundary in twin G2. Their stress, strain, and plastic deformation gradients are also low in the final state. Moreover, the stress values at these two points decrease to different degrees at strains of 32% and 36%, respectively. The stress at point G decreases by 27 MPa, and the amplitude decreases by approximately 4%. The final stress at point H is only 550 MPa. Combined with the quasi in situ tensile test results, the Σ3 boundaries near the four points in Figure 9 all exceed the Brandon criterion during plastic deformation. This indicates that in the process of plastic deformation, Σ3 boundaries can release the surrounding stress, thus alleviating the stress concentration inside the material, which is conducive to a great degree of plastic deformation.

**Figure 10:**
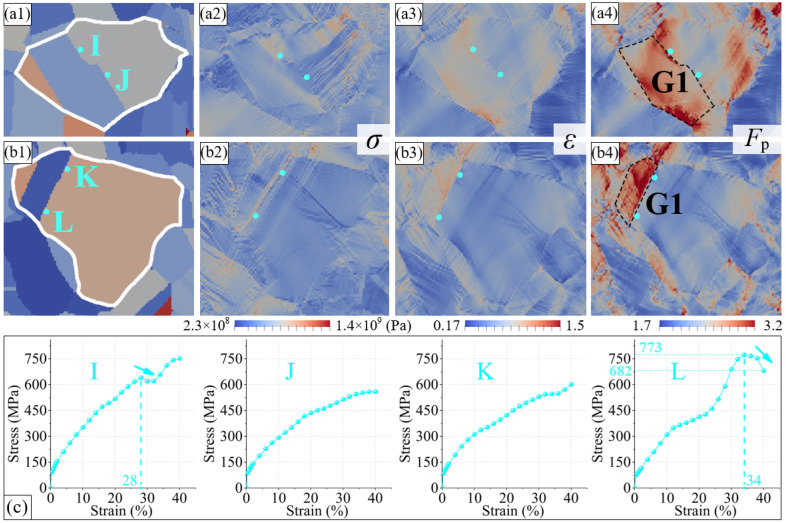
(**a1**) The position of points near Σ3 boundaries at initial simulation state in grain group GGⅢ. (**a2**–**a4**) The distribution of stress, strain, and plastic deformation gradient at final simulation state in grain group GGⅢ. (**b1**) The position of points near Σ3 boundaries at initial simulation state in grain group GGⅣ. (**b2**–**b4**) The distribution of stress, strain, and plastic deformation gradient at final simulation state in grain group GGⅣ. (**c**) The stress variation of points I–L.

The quasi in situ tensile test proved that a part of the Σ3 boundaries were always stable during the plastic deformation process, and the stress state around them was analyzed in detail using CPFE simulation. Figure 10 shows the stress and strain distributions at the final state in the GGⅢ and GGⅣ. In the figure, four points (I, J, K, and L) are located outside of twins, which are surrounded by Σ3 boundaries. Compared with the stress near the random boundaries in Figure 8, the stress at the four points in Figure 10 is noticeably lower. The phenomenon of the decreasing stress with the increasing strain is also observed at points I and L, and the reduction amplitude at point L reaches 12%. However, the stresses at points J and K maintain continuous growth. This shows that Σ3 boundaries may alleviate the surrounding stress concentration under the premise of maintaining a good mirror symmetry relationship. In addition, if theΣ3 boundary outside a twin does satisfy the Brandon criterion, the plastic deformation gradient within the twin is generally small, such as twin G1 in Figure 9(a4) and twin G2 in Figure 9(b4). In contrast, the twins surrounded by the Σ3 boundary have a high plastic deformation gradient, such as twin G1 in Figure 10(a4) and twin G1 in Figure 10(b4). This indicates that twins can also have good plastic deformation coordination, which positively affects the plasticity.

### 4.2. Mechanism of the Effect of GB Types on Dislocations and Plasticity

The microscopic essence of plastic deformation involves a dislocation slip and twinning. In fcc metals, the slip is dominant. When the applied stress exceeds the elastic limit, a crystal begins to undergo plastic deformation, and the slip system {111}<110> rapidly begins, together with dislocation proliferation and diffusion. Some dislocations move to the GBs, some pass directly through the GB, some are reflected into the grain, and many are blocked by the GBs, resulting in stress concentration. As a result of the repulsive force between dislocations, further movement of the dislocations within the grains is inhibited when the dislocation pile-up is severe, preventing further plastic deformation. This stress at the GB must be released. Studies have shown that there are two primary methods of stress release at GBs: core delocalization or incorporation within the interface structure [16]. The relaxation is closely related to the GB type, irrespective of the mode.

Pure nickel is a typical fcc metal with {111} slip and twin planes. Many annealing twins and Σ3 twin boundaries were formed in the nickel samples after GB modification; that is, many regularly arranged {111} planes were formed within them. Simultaneously, there were also many random boundaries; that is, ordinary high-angle boundaries. Although the packing density of a random boundary is lower than a Σ3 boundary, its atomic arrangement is much more disordered. Thus, the random boundary has higher GB energy than the Σ3 boundary. The experiments and simulations in this study show that with the increase in plastic deformation, the dislocation density near the random boundaries increases (Figure 6), and the stress increases gradually (Figure 8). These trends indicate that a random boundary has a strong hindrance effect on the dislocations, which means that a dislocation slip is difficult to achieve.

Σ3 twin boundaries in pure nickel were formed through rotating the fcc lattice around the <111> axis by 60°. The coherent Σ3_c_ boundary was located on the {111} plane, and the {111} planes on both sides were parallel to each other, which is a prerequisite for the direct transmission of dislocations [16,38]. Figure 5(c3,d3) shows that in the grain groups GGⅢ and GGⅣ, the slip traces on both sides of the Σ3 boundary are parallel to each other, indicating that a dislocation slip can pass through the Σ3 twin boundary and that such direct transmission may occur. According to the study by Z.-H. Jin [23,24], when a common non-screw dislocation in fcc metals is forced by an external stress into a coherent twin boundary, it can dissociate into different partial dislocations, gliding into the twin, as well as along the twin boundary. A screw dislocation may either propagate into the adjacent twin by cutting through the boundary or it may dissociate within the boundary plane. This makes the slip transmission much easier. The incoherent Σ3_ic_ twin boundary is not on the {111} plane. In most cases, it is parallel to the {112} plane. When the dislocation slips to the Σ3_ic_ boundary, indirect slip transmission is more likely to occur. The Σ3_ic_ boundary consists of a series of Shockley partial dislocations on every {111} plane with a repeatable sequence of *b*_2_:*b*_1_:*b*_3_ [39]. Dislocation *b*_1_ is a pure edge dislocation with a Burgers vector of 1/6[11−2]. The Burgers vectors of *b*_2_ and *b*_3_ are 1/6[−211] and 1/6[1−21], respectively, which are mixed partial dislocations with screw components. Shockley partial dislocations can slip flexibly on the {111} plane to meet and react with other dislocations piled near the Σ3_ic_ boundaries. Therefore, it is speculated that the dislocation reactions may occur when the intragranular dislocations move into the Σ3 boundaries, resulting in annihilation, dissociation, or other phenomena. Thus, the number of dislocations near the GB and the corresponding stress concentration can be reduced. This may be one of the reasons for the stress release near the Σ3 boundary in the experiments and simulations conducted in this study. In addition, the simulation results in Figure 9 and Figure 10 show that the stress reduction near the Σ3 boundary occurs after the stress and strain has accumulated to a specific extent, which would not occur at low stress and strain. According to the quasi in situ tensile test, the identification of a Σ3 boundary as a random boundary also occurred at high strain conditions (Figure 6). Moreover, the symmetric twin structures of Σ3_ic_ boundaries may be destroyed after a series of dislocation reactions. This indicates that the type of transformation or identification error of the Σ3 boundary is closely related to the stress-releasing phenomenon. Furthermore, this phenomenon has a specific threshold, possibly because some dislocation reactions must be activated by a critical stress, which is worthy of further investigation.

In conclusion, both the Σ3_c_ and Σ3_ic_ boundaries have a significantly smaller hindering effect on the dislocation slip than the random boundaries, and the Σ3 boundary can release specific internal stresses. Therefore, in GB modification, stimulating Σ3 boundaries and reducing the proportion of random boundaries can effectively improve the plasticity of the material. It should be noted that the Σ3 boundary may also be composed of a series of small facets. The interactions between them and the dislocations may have some differences and a higher temperature may cause a faceting–roughening transition. This will influence the plastic deformation and should be further explored in future research.

## 5. Conclusions

In this study, quasi-in situ tensile tests and the corresponding CPFE simulations were performed on a pure nickel wire undergoing GB modification. The evolution of the grain orientation, activation of the slip system, and relationship between the dislocations and GB during plastic deformation were analyzed. In addition, the positive effect of the Σ3 boundary on the plastic deformation owing to GB modification was demonstrated. The following are the primary conclusions:

1. The fraction of Σ3 boundaries decreases gradually during tensile deformation. Most grains rotate in the direction of the [001]–[111] line link. The rotation amplitude is affected by the initial orientation of the grain. Twins can also rotate. However, the rotation amplitude is typically less than that of other grains. Some twins surrounded by Σ3 boundaries may exhibit a good deformation coordination.
2. The phenomenon of fragmentation into sub-grains common occurs in large grains, while it is rare in twins with Σ3 boundaries. Even if the orientation of a sub-grain is completely different from that of the matrix grain, it can still maintain the dominant slip system of the matrix grain in the subsequent deformation process. The Schmid and geometrical compatibility factors jointly determine the actual activated slip system, and the Schmid factor plays the predominant role. With an increase in strain, the slip system corresponding to the maximum Schmid factor of each grain and the actual active slip system remain unchanged. The (111) and (1−1−1) slip planes are the most active slip systems.
3. In the plastic deformation process, the random boundary has a strong hindrance effect on the dislocations. The stress near it continues to accumulate, which may cause microcracks. Conversely, the Σ3 boundary shows a weaker hindrance effect on the dislocations, which is conducive to the plasticity of materials. The primary reasons may be as follows: Firstly, the parallel slip planes on both sides of the coherent Σ3c boundary provided a good prerequisite for the direct slip transmission of the dislocations. Secondly, the incoherent Σ3ic boundary is supposed to release the surrounding stress concentration through dislocation reactions. However, the occurrence of stress release had a particular threshold. Thirdly, the geometrical compatibility factor of some Σ3 boundaries improved with an increase in strain, thus enhancing the deformation coordination of the grains on both sides.

## Figures and Tables

**Table 1:** The absolute value of Schmid factors at a strain of 19.8%.

Grain ID		Slip System											
		S1	S2	S3	S4	S5	S6	S7	S8	S9	S10	S11	S12
		(111)			(-1-11)			(1-1-1)			(-11-1)		
		[01-1]	[-101]	[1-10]	[0-1-1]	[101]	[-110]	[0-11]	[-10-1]	[110]	[011]	[10-1]	[-1-10]
GGⅠ	G1	0.005	0.177	0.182	0.301	0.040	**0.341**	0.274	0.045	0.319	0.022	0.097	0.119
	G2	0.189	0.274	**0.463**	0.397	0.181	0.215	0.095	0.008	0.090	0.302	0.368	0.066
	G3	0.029	0.212	0.184	0.208	0.040	0.248	0.355	0.012	**0.367**	0.119	0.143	0.024
GGⅡ	G1	0.329	**0.424**	0.094	0.007	0.065	0.058	0.091	0.264	0.174	0.232	0.333	0.101
	G2	0.271	**0.486**	0.215	0.021	0.145	0.124	0.114	0.415	0.301	0.177	0.372	0.194
	G3	0.189	0.491	0.303	0.086	0.304	0.218	0.192	**0.493**	0.301	0.083	0.300	0.217
	G4	0.288	**0.425**	0.137	~0	0.003	0.003	0.035	0.291	0.270	0.254	0.391	0.137
GGⅢ	G1	0.457	0.096	0.361	0.224	0.259	0.035	**0.490**	0.198	0.292	0.256	0.394	0.137
	G2	0.280	0.215	**0.495**	0.292	0.201	0.091	0.275	0.079	0.196	0.287	0.490	0.203
	G3	0.212	0.281	**0.494**	0.341	0.184	0.157	0.150	0.029	0.122	0.279	0.432	0.153
GGⅣ	G1	0.058	0.216	0.158	0.111	0.184	0.295	0.257	0.242	**0.499**	0.204	0.474	0.270
	G2	0.225	**0.497**	0.272	0.074	0.263	0.189	0.203	0.487	0.284	0.096	0.294	0.198
	G3	0.230	**0.499**	0.269	0.050	0.238	0.188	0.154	0.468	0.314	0.126	0.345	0.219

Note: The maximum Schmid factor corresponding to each grain is shown in bold, and the Schmid factor corresponding to the actual activated slip systems is marked in blue.

**Table 2:** *m*′ values of several grains with different slip systems at strain values of 19.8%.

Grain ID		Slip System											
		S1	S2	S3	S4	S5	S6	S7	S8	S9	S10	S11	S12
GGⅡ	G1 and G3	0.003	0.475	0.478	0.161	0.285	0.446	0.224	0.400	**0.624**	0.002	0.191	0.193
GGⅣ	G1 and G2	0.472	**0.999**	0.527	0.165	0.172	0.337	0.152	0.012	0.164	0.166	0.179	0.013

Note: G1 and G2 represents the *m*′ between the slip system in grain G1 with the maximum Schmidt factor and the 12 slip systems in grain G2. The maximum *m*′ corresponding to each group is shown in bold, and the *m*′ corresponding to the actual activated slip system is marked in blue.

**Table 3:** The variation of *m*′ values of several grains during tensile test.

Grain ID	Strain (%)	Slip System											
		S1	S2	S3	S4	S5	S6	S7	S8	S9	S10	S11	S12
GGⅡ−G1 and G2	0	0.011	0.464	0.457	0.193	0.284	0.477	0.264	0.374	0.638	0.007	0.172	0.165
	10.5	0.052	0.461	0.409	0.222	0.312	0.535	0.277	0.315	0.592	0.037	0.192	0.155
	19.8	0.072	0.435	0.364	0.263	0.325	0.588	0.307	0.266	0.573	0.056	0.186	0.131
	29.6	0.076	0.404	0.328	0.290	0.336	0.626	0.322	0.242	0.564	0.063	0.180	0.117
GGⅢ−G1 and G2	0	0.440	0.556	0.997	0.195	0.004	0.199	0.185	0.153	0.338	0.134	0.004	0.137
	10.5	0.472	0.527	0.999	0.183	~0	0.183	0.180	0.161	0.341	0.147	~0	0.147
	19.8	0.536	0.433	0.969	0.225	0.057	0.169	0.268	0.181	0.449	0.079	0.021	0.057
	29.6	0.526	0.464	0.990	0.191	0.028	0.163	0.228	0.188	0.415	0.120	0.018	0.102
GGⅤ−G1 and G2	0	0.389	0.626	0.236	0.457	0.009	0.448	0.477	0.3	0.177	0.181	0.006	0.187
	10.5	0.368	0.633	0.265	0.453	0.018	0.436	0.495	0.295	0.12	0.162	~0	0.173
	19.8	0.235	0.536	0.301	0.531	0.102	0.429	0.524	~0	0.237	0.139	0.075	~0
	29.6	0.224	0.056	0.172	0.37	0.434	0.064	0.552	0.241	0.312	0.359	0.57	0.212

Note: The *m*′ corresponding to the actual activated slip system is marked in blue.

## Data Availability

Not applicable.
